# Gene Expression Changes in Phosphorus Deficient Potato (*Solanum tuberosum* L.) Leaves and the Potential for Diagnostic Gene Expression Markers

**DOI:** 10.1371/journal.pone.0024606

**Published:** 2011-09-14

**Authors:** John P. Hammond, Martin R. Broadley, Helen C. Bowen, William P. Spracklen, Rory M. Hayden, Philip J. White

**Affiliations:** 1 Division of Plant and Crop Sciences, University of Nottingham, Loughborough, United Kingdom; 2 Warwick HRI, University of Warwick, Wellesbourne, Warwick, United Kingdom; 3 The James Hutton Institute, Invergowrie, Dundee, United Kingdom; United States Department of Agriculture, Agricultural Research Service, United States of America

## Abstract

**Background:**

There are compelling economic and environmental reasons to reduce our reliance on inorganic phosphate (Pi) fertilisers. Better management of Pi fertiliser applications is one option to improve the efficiency of Pi fertiliser use, whilst maintaining crop yields. Application rates of Pi fertilisers are traditionally determined from analyses of soil or plant tissues. Alternatively, diagnostic genes with altered expression under Pi limiting conditions that suggest a physiological requirement for Pi fertilisation, could be used to manage Pifertiliser applications, and might be more precise than indirect measurements of soil or tissue samples.

**Results:**

We grew potato (*Solanum tuberosum* L.) plants hydroponically, under glasshouse conditions, to control their nutrient status accurately. Samples of total leaf RNA taken periodically after Pi was removed from the nutrient solution were labelled and hybridised to potato oligonucleotide arrays. A total of 1,659 genes were significantly differentially expressed following Pi withdrawal. These included genes that encode proteins involved in lipid, protein, and carbohydrate metabolism, characteristic of Pi deficient leaves and included potential novel roles for genes encoding patatin like proteins in potatoes. The array data were analysed using a support vector machine algorithm to identify groups of genes that could predict the Pi status of the crop. These groups of diagnostic genes were tested using field grown potatoes that had either been fertilised or unfertilised. A group of 200 genes could correctly predict the Pi status of field grown potatoes.

**Conclusions:**

This paper provides a proof-of-concept demonstration for using microarrays and class prediction tools to predict the Pi status of a field grown potato crop. There is potential to develop this technology for other biotic and abiotic stresses in field grown crops. Ultimately, a better understanding of crop stresses may improve our management of the crop, improving the sustainability of agriculture.

## Introduction

Phosphorus (P) is an essential macronutrient required by all living organisms, but it is also one of the most unavailable and inaccessible nutrients present in the soil. Thus, establishing a plant's requirement for P, and the ability of the soil to supply this requirement in the form of phosphate (Pi), is crucial in maintaining crop yield and quality, and avoiding excessive P entering the environment, where it can contribute to the process of eutrophication [1]–[4]. Also, since over 85% of mined P is used in food production [5] and consumption of this non-renewable resource will lead to peak phosphorus production (akin to peak oil) [6], [7], there will be increasing pressures on Pi fertiliser availabilities and costs in the future. These pressures will be exacerbated by increasing demand on food production systems as the human population increases and by fluctuation in oil prices [7]. Therefore, a better understanding of how plants respond to Pi availability and the factors affecting the availability of soil Pi to plants will optimise additions of Pi fertilisers, allowing a balance to be reached whereby the available Pi matches the crop's requirements [8], [9].

In many plants, a prolonged reduction in the availability of soil Pi reduces their yield. However, plants have evolved to cope with poor Pi availability in soils, and the mechanisms they have developed act both to increase acquisition of Pi from the soil and to improve its use internally [3], [10]–[15]. Visually, Pi deficiency results in a reduced stature, acute leaf angles, suppression of tillering, prolonged dormancy, early senescence and decreased size and number of flowers and buds [16]. The development of dark green or blue-green foliage is among the first symptoms of Pi deficiency. Red, purple or brown pigments develop in leaves, especially along veins. This is a consequence of anthocyanin production, which is induced by increased leaf sucrose concentrations [17]–[20] and is thought to protect nucleic acids from UV damage and chloroplasts from photoinhibitory damage caused by P-limited photosynthesis [21].

The majority of these visual symptoms are not apparent until Pi deficiency is advanced. Consequently, remedial action to correct advanced Pi deficiency, diagnosed by visual symptoms, is often too late to overcome the yield reduction. Thus, *a priori* knowledge of the soil's ability to supply adequate amounts of Pi or the Pi status of the crop is required to manage crop P nutrition effectively by applying inorganic Pi fertilisers. This maintains an adequate supply of Pi to the roots of crop plants, thereby ensuring crop yield and quality is maintained. This is traditionally assessed through analysis of soil or plant tissue for their P or Pi content [22]. The regression of the soil or plant Pi content against crop yield, using mathematical equations or models, can then be used empirically to determine the Pi levels required to obtain maximum yields. Calibrating these mathematical equations and models is a major investment for establishing soil and plant tests, since they must be calibrated against many crops, soil types and geo-climatic regions [23]. Alternatively, using transcriptional profiling technology, it may be possible to assess the physiological Pi status of a plant through the analysis of transcribed genes [22], [24].

The use of transcriptional profiling is now widespread in biology and has been employed to characterise the transcriptional profiles of plants during development and under biotic and abiotic stresses, including Pi deficiency [24]–[39]. These data have shown, across plant species, characteristic transcriptional events in response to Pi deficiency. These include changes in the expression of genes involved in remobilizing, decreasing or replacing Pi in non-essential cellular compounds, the exudation of metabolites and enzymes into the rhizosphere to increase soil Pi availability, changes in root morphology and/or associations with micro-organisms and increased capacity to acquire Pi more effectively from the soil. Therefore, it might be possible to associate these characteristic responses to Pi deficiency with plant physiological Pi status.

Here we use an oligonucleotide potato microarray [40] to investigate the transcriptional profile of potato leaves under Pi deficiency and compare this with previously described transcriptional profiles from the leaves of Arabidopsis and rice. We identify novel components to these profiles, including the increased expression of potato patatin genes, with potential phospholipase A2 activity, in the leaves of Pi deficient potatoes. As a proof-of-concept, we use these data to test if diagnostic gene expression markers for Pi deficiency in potato leaves can be identified and used to predict the Pi status of field grown potato plants. A set of 200 diagnostic genes were identified that could distinguish between fertilised and unfertilised potato plants. There is potential to use this technology for optimising Pi fertiliser applications to potatoes and it may be applicable to other nutrient, abiotic or biotic stresses.

## Results and Discussion

### Leaf [P] declines rapidly after Pi withdrawal

**Figure 1:**
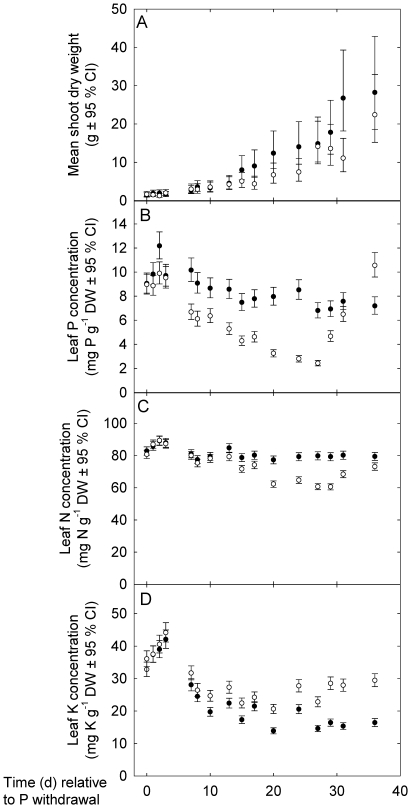
Biomass and macronutrient content of hydroponically grown potato plants. Mean shoot dry weights (A), leaf P concentrations (B), leaf N concentrations (C) and leaf K concentrations of potato plants supplied with a full nutrient solution (closed circles) or supplied with a nutrient solution containing no Pi (open circles). Plants were grown in an NFT hydroponic system under glasshouse conditions. Pi was withdrawn from the nutrient solution supplying half the plants for 28 d before being re-supplied. Data points represent mean ±95% confidence interval (n = 6).

To induce Pi deficiency symptoms, potato plants were grown under glasshouse conditions using a nutrient film technique (NFT) hydroponic system to control their nutrient status accurately. The shoot dry weights of plants supplied with nutrient solution containing no Pi were not significantly different from the shoot dry weights of plants supplied with a full nutrient solution up to 13 d after the withdrawal of Pi (Figure 1A). The shoot dry weights of plants supplied with nutrient solution containing no Pi were significantly (*P*<0.05) reduced compared with plants supplied with a full nutrient solution 15, 17 and 24 d after the withdrawal of P. The delayed impact of the withdrawal of Pi from the nutrient solution on shoot dry weight has been observed previously in Arabidopsis [24], [30], maize [35] and rice [28].

The effect of Pi withdrawal on the leaf phosphorus concentration (leaf [P]) was rapid (Figure 1B). Leaf [P] of plants supplied with nutrient solution containing no Pi was significantly (*P*<0.05) less than that of plants supplied with a full nutrient solution one day after the withdrawal of Pi from the nutrient solution. A reduced leaf [P] in plants supplied with nutrient solution containing no Pi was apparent until three days after the re-supply of Pi to the nutrient solution, when leaf [P] of plants supplied with nutrient solution containing no Pi had increased back to a similar leaf [P] of plants supplied with a full nutrient solution. The leaf [P] of plants supplied with nutrient solution containing no Pi continued to increase after the re-supply of Pi and was significantly (*P*<0.05) greater than that of plants supplied with a full nutrient solution seven days after the re-supply of Pi to the plants (Figure 1B). This is consistent with an increased potential to acquire Pi from the nutrient solution, established during Pi deficiency, allowing greater uptake of Pi after its re-supply to the nutrient solution [19], [41].

The effect of Pi withdrawal on leaf nitrogen (N) and potassium (K) concentrations was also monitored (Figure 1, C and D). Leaf N concentrations (mg N g^−1^ DW) of plants supplied with nutrient solution containing no Pi were significantly (*P*<0.05) less than those of plants supplied with a full nutrient solution 13 d after the withdrawal of Pi from the nutrient solution until the end of the experiment. Leaf K concentrations (mg K g^−1^ DW) of plants supplied with nutrient solution containing no Pi were significantly (*P*<0.05) greater than those of plants supplied with a full nutrient solution 13 d after the withdrawal of Pi from the nutrient solution until the end of the experiment.

### Changes in Pi supply induce rapid changes in gene expression

To identify genes that respond specifically and rapidly to Pi deficiency, total RNA was extracted from the diagnostic leaves of potato plants grown in a NFT hydroponic system in the glasshouse. Total RNA was labelled and hybridised to the Potato Oligo Chip Initiative (POCI) arrays to determine the expression levels of 42,034 potato genes [40]. A pre-filtered gene list was used to identify genes whose expression was significantly up- or down-regulated in at least two of the seven time points. A total of 1,659 genes were significantly (*P*<0.01) differentially expressed, with 762 genes up-regulated by more than 1.5 fold at two or more of the seven time points and 965 genes down-regulated by more than 1.5 fold in two or more of the seven time points (Table S1). Following hierarchical clustering [42] of the significantly differentially expressed genes, eight distinct patterns of gene expression (Group I–VIII) were identified after the withdrawal and subsequent re-supply of Pi to the nutrient solution (Table 1; Figure S1).

**Table 1:** Summary of groups identified following hierarchical clustering of expression data.

Group^a^	Expression profile^b^							Number of genes	GO Terms^c^
	0	1	3	7	17	29	31		
									GO:30414: protease inhibitor activity
									GO:4866: endopeptidase inhibitor activity
I	**-**	**-**	**-**	**-**	**↑**	**↑**	**-**	396	GO:4857: enzyme inhibitor activity
									GO:30234: enzyme regulator activity
									GO:4867: serine-type endopeptidase inhibitor activity
									GO:3676: nucleic acid binding
									GO:3735: structural constituent of ribosome
II	**-**	**-**	**-**	**-**	**↓**	**↑**	**-**	255	GO:5198: structural molecule activity
									GO:3723: RNA binding
									GO:8026: ATP-dependent helicase activity
									GO:3824: catalytic activity
									GO:16787: hydrolase activity
III	**↓**	**-**	**-**	**-**	**-**	**-**	**-**	301	GO:45156: electron transporter, in the cyclic electron transport pathway of photosynthesis
									GO:30554: adenyl nucleotide binding
									GO:17076: purine nucleotide binding
									GO:46857: oxidoreductase activity, with NAD or NADP as acceptor
									GO:16661: oxidoreductase activity
IV	**-**	**-**	**-**	**-**	**-**	**-**	**-**	158	GO:20037: heme binding
									GO:46906: tetrapyrrole binding
									GO:5506: iron ion binding
									GO:50589: leucocyanidin oxygenase activity
V	**-**	**-**	**-**	**-**	**↓**	**↓**	**-**	211	GO:16210: naringenin-chalcone synthase activity
									GO:4097: catechol oxidase activity
									GO:5198: structural molecule activity
									GO:4871: signal transducer activity
									GO:4672: protein kinase activity
VI	**-**	**-**	**↑**	**↑**	**-**	**-**	**-**	223	GO:4872: receptor activity
									GO:3700: transcription factor activity
									GO:4674: protein serine/threonine kinase activity
									GO:8061: chitin binding
									GO:1871: pattern binding
VII	**-**	**-**	**-**	**↑**	**↑**	**-**	**-**	85	GO:30247: polysaccharide binding
									GO:4568: chitinase activity
									GO:30246: carbohydrate binding
									GO:4289: subtilase activity
VIII	**-**	**-**	**-**	**↑**	**-**	**-**	**-**	30	GO:42802: protein self binding
									GO:4252: serine-type endopeptidase activity

a distinct patterns of gene expression were identified following hierarchical clustering [42] of the significantly differentially expressed genes (Figure S1).

b Average expression profiles were calculated in GeneSpring for each group of genes across the time series. Column headers represent time (d) relative to Pi withdrawal, with Pi being re-supplied after 28 d. Symbols: ↑ average group gene expression up-regulated more than 1.5-fold, ↓ average group gene expression down-regulated more than 1.5-fold, - average group gene expression between 1.5 down-regulated and 1.5-fold up-regulated, i.e. no change.

c Top five Gene Ontology terms present in each group that appear more often than would be expected by chance, identified using GeneSpring's GO Ontology Browser.

Changes in the abundance of transcripts on day 0, occurred 2 hours after the change in Pi supply. At day 0, the transcript abundance of 272 genes was significantly greater and the transcript abundance of 475 genes was significantly lower in plants 2 h after the withdrawal of Pi from the nutrient solution, compared to their abundance in plants supplied with the full nutrient solution (Table S1). Transcripts with greater abundance at day 0 were mainly clustered with Groups I and VI, where the average gene expression profile decreased between day 0 and 1 (Table 1; Figure S1). Similarly, transcripts with less abundance at day 0 were mainly clustered with Groups II and III, where the average gene expression profile increased between day 0 and 1 (Table 1; Figure S1). These represent transient changes in the abundance of these transcripts and are consistent with previous observations for early non-specific changes in gene expression following the withdrawal of Pi from the nutrient solution [24].

### Protein synthesis and DNA replication are altered by Pi deficiency

Group I genes had greater transcript abundance 17 d after the withdrawal of Pi from the nutrient solution. The high average expression was also sustained one day after Pi was re-supplied to the nutrient solution of plants previously supplied with no P, compared with leaves from plants supplied with a full nutrient solution (Table 1; Figure S1). This group of genes contained a greater number of genes that had been annotated with Gene Ontology (GO) terms for enzyme inhibitor activity than would be expected by chance. The genes annotated with enzyme inhibitor activity included several genes with aspartic and cysteine protease inhibitor activity, suggesting a reduction in protein catabolism. There were also several genes annotated with protease activity, suggesting protein recycling mechanisms had been activated to recycle amino acids. This was supported by the general down-regulation of genes involved in the synthesis of amino acids (Table S1).

Transcript abundance increased rapidly and specifically after changes to the availability of Pi in the nutrient solution on day 29 for genes in Group II, with transcript levels decreasing significantly up to this point (Table 1; Figure S1). Of the 255 genes in Group II, 32 had homology to ribosomal protein genes, peptidylprolyl isomerases, or eukaryotic translation initiation factors, suggesting protein synthesis is suppressed during Pi deficiency and a rapid increase upon the re-supply of Pi to the nutrient solution.

Increased recycling of proteins and altered amino acid biosynthesis has previously been reported in Pi deficient leaves [24], [30], [31], [33], [43]. Hammond et al. [24] previously reported an increase in the number of genes annotated with gene ontology terms for protein fate, whilst Wu et al. [30] and Misson et al. [31] both observed increased expression of genes involved in protein degradation and the suppression of genes involved in protein biosynthesis in the leaves of Arabidopsis plants during Pi starvation.

Genes clustered in Group II shared several functional groupings with genes in Group V. The transcript abundance of Group V genes decreased with increasing Pi deficiency, but their abundance was slower to return to basal levels upon re-supply of Pi to the nutrient solution. In Group II, 22 genes had homology with proteins involved in chromatin structure and DNA replication, including seven helicases, three histone deacetylases and two matrix attachment region proteins. Of the 211 Group V genes, 18 had homology to genes encoding all major classes of histone proteins, and a further seven had homology to ribosomal protein genes. A putative role for histone H2A.Z has recently been proposed in the transcriptional suppression of genes that respond to Pi deficiency in plants [44]. The down-regulation of genes involved in histone formation supports the involvement of these proteins in regulating some responses of plants to Pi deficiency. Genes clustered in Group V also had homology to genes involved in cell wall biosynthesis, including extensin-like proteins, pectin methylesterases, polygalacturonases, and cell cycle regulation, including cyclins, cyclin-dependent protein kinases and cyclin-dependent protein kinase regulators. These expression profiles suggest an overall down regulation of DNA replication, chromatin formation, and cell cycle progression associated with severe Pi deficiency. This may be a consequence of reduced cellular development in the leaves of Pi deficient potatoes, as more C is allocated to the roots [45]. Reduced cell cycle activity has previously been observed in Pi starved cells and plants [46], [47]. These two clusters also contain genes involved in purine and pyrimidine biosynthesis, whose transcript abundance decreased after the withdrawal of P. Nucleic acids represent a valuable source of P in cells that can be redirected under Pi deficiency [48]. The down regulation of the purine and pyrimidine biosynthetic pathways was observed in Arabidopsis leaves, along with the induction of genes involved in nucleotide breakdown [31], [33]. Another GO term overrepresented in these clusters was nucleotide binding, which included proteins capable of binding DNA, RNA and ATP.

### Reduced expression of chlorophyll and anthocyanin biosynthesis genes

**Figure 2:**
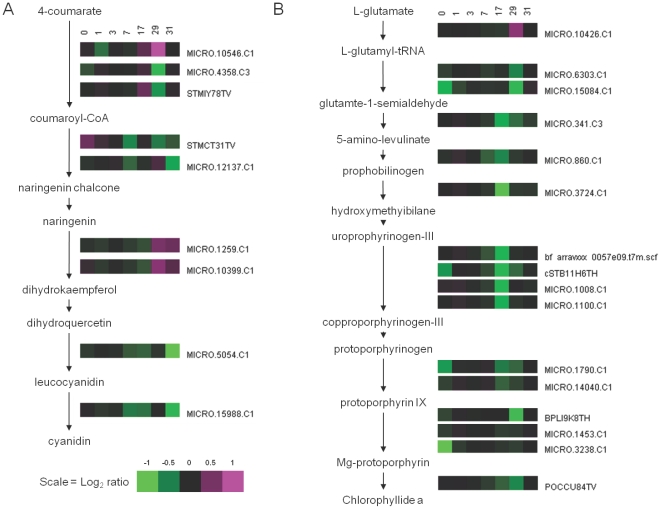
Anthocyanin and chlorophyll pathway analysis. Expression profiles of transcripts with homology to genes that encode proteins involved in (A) anthocyanin biosynthesis and (B) chlorophyll biosynthesis pathways. Pathways were populated with genes using Solanaceae Genomics Network Unigene identifiers and the PotatoCyc pathway tool (http://solcyc.solgenomics.net/expression.html). Numbers above heatmaps are time-points relative to the withdrawal of Pi from the nutrient solution (d), with Pi being re-supplied to the nutrient solution after 28 d.

A common physiological response to Pi deficiency is the development of red, purple or brown pigments in the leaves, especially along veins. This is a consequence of anthocyanin production, which is probably induced by increased leaf sucrose concentrations [17]–[20] and is thought to protect nucleic acids from UV damage and chloroplasts from photoinhibitory damage caused by P-limited photosynthesis [21]. This has been observed at the transcriptional level, with the increased expression of genes involved in the biosynthesis of anthocyanins and their precursors [24], [31], [33]. Curiously, a number of genes involved in the biosynthesis of anthocyanins and their precursors were clustered in Group V, and their transcript abundance was decreased under Pi deficient conditions. These included a 1.7-fold repression of a leucoanthocyanidin dioxygenase and a 1.6-fold repression of a dihydroflavonol 4-reductase, 17 d after the withdrawal of Pi from the nutrient solution (Figure 2A). Visible anthocyanin accumulation was not observed in the leaves of potatoes grown under Pi deficient conditions, but was observed in the stem tissues of potato plants not supplied with Pi in the nutrient solution. This is consistent with previous descriptions of visual Pi deficiency symptoms in potato [49], [50].

Phosphate starvation has direct consequences for photosynthesis and C assimilation [45], [48], [51]. Excess Pi taken up by plants, is stored in the vacuole and used to maintain cellular Pi homeostasis [3], [52], [53]. In Pi deficient plants, when the available vacuolar Pi reserves are depleted, the lack of cytoplasmic Pi often inhibits photosynthesis, although some research suggests that photosynthesis can be sustained under Pi deficiency for some time [54]. As a consequence, chlorosis was not observed during Pi starvation, in contrast to K and Mg starvation [55]. However, the reduction in photosynthesis was evident in the down regulation of many genes encoding proteins involved in photosynthesis, including photosystem subunits I and II, small subunits of RuBisCo and chlorophyll biosynthesis [30], [33]. In the leaves of Pi deficient potato there was a gradual decline in the abundance of transcripts with homology to genes involved in the biosynthesis of chlorophyllide a, a precursor for chlorophyll synthesis (Figure 2B). The abundance of many of the transcripts in this pathway was also slow to return to basal levels, remaining suppressed for 24 h after the re-supply of Pi to the nutrient solution. Morcuende et al. [33] attributed this slow reversal in transcriptional repression of photosynthesis to the secondary nature of this response, linked to decreased photosynthetic demand and accumulation of sugars during Pi deficiency.

Although carbon assimilation is restricted during Pi starvation and the export of triose-Pi from the chloroplast is reduced, resulting in its conversion to starch, a sustained, and in some species an increased translocation of mobile carbohydrates via the phloem to the roots is observed, primarily in the form of sucrose [43], [48], [51], [56]. The transcript abundance of many genes associated with carbohydrate metabolism fluctuated greatly during the time course of the experiment (Table S1). This included the 14-fold increase in the transcript abundance of a starch synthase, a 10-fold increase in the transcript abundance of a PEP carboxylase kinase, an 8-fold increase in the transcript abundance of a PEPCase, and a 6.5-fold increase in the transcript abundance of a phosphofructokinase (Table S1). Their increased expression in Pi deficient leaves of potato was consistent with previous studies in Arabidopsis and rice [24], [29], [30], [33], [34], and supports the altered flux of carbohydrates through glycolysis and increase in starch synthesis.

### Membrane lipid synthesis and catabolism modulated by leaf Pi status

**Figure 3:**
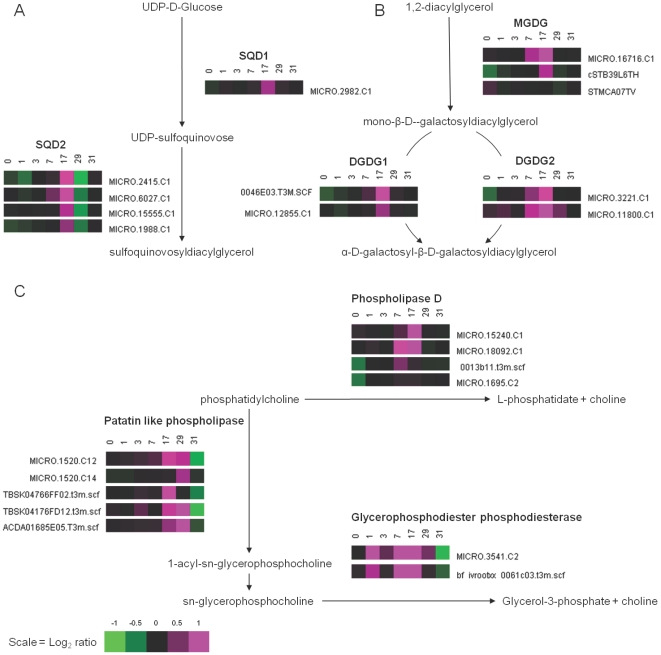
Pathway analysis for sulpho- and galacto-lipid biosynthesis and phospholipid degradation. Expression profiles of transcripts with homology to genes that encode proteins involved in (A) sulpholipid biosynthesis, (B) galactolipid biosynthesis and (C) phospholipid degradation pathways. Pathways were populated with genes using Solanaceae Genomics Network Unigene identifiers and the PotatoCyc pathway tool (http://solcyc.solgenomics.net/expression.html). Numbers above heatmaps are time-points relative to the withdrawal of Pi from the nutrient solution (d), with Pi being re-supplied to the nutrient solution after 28 d.

Metabolic and biochemical reactions either releasing P from metabolites or conserving P through alternative metabolic reactions were represented by many genes in Group VII, whose expression increased up to day 17, before rapidly returning to basal levels after the re-supply of Pi to the nutrient solution. These included many genes involved in lipid metabolism (Figure 3). The polar lipids that make up cell and organelle membranes consist of a mixture of phospho-, galacto- and sulpho-lipids, with galactolipids constituting up to 70% of membrane lipids in plant leaves, with the remainder represented by phospholipids [57], [58]. Under Pi limiting conditions, plants can alter the composition of their membranes, reducing their phospholipid content by replacing phospholipids with galacto- and sulpho-lipids [59]–[62], a process that may be regulated through crosstalk between the auxin and cytokinin signalling pathways [63]. These compositional changes are effected through the synthesis of new galacto- and sulpho-lipids and the regulated breakdown of some phospholipids to release P to maintain cellular Pi homeostasis and diacylglycerol groups for galacto- and sulpho-lipid synthesis [59]–[62], [64]–[66]. In the leaves of potato, we observed the increased abundance of transcripts encoding enzymes involved in galacto- and sulpho-lipid biosynthesis (Figure 3AB). Specifically, the abundance of transcripts encoding the sulpholipid biosynthesis genes, *SQD1* and *SQD2*, increased 7 d after the withdrawal of Pi from the nutrient solution, with an average fold change of 1.2 for treated versus control plants. The abundance of these transcripts continued to increase up to 17 d after the withdrawal of Pi from the nutrient solution, with an average fold change of 2.4 for treated versus control plants (Figure 3A). For galacto-lipid biosynthesis, the abundance of transcripts encoding MGDG, DGDG1, and DGDG2 followed a similar pattern, increasing 7 d after the withdrawal of Pi from the nutrient solution in treated plants up to 17 d after the withdrawal of Pi from the nutrient solution, with an average fold change of 3.2 for treated versus control plants (Figure 3B). Similar expression profiles have been observed previously for these genes in Arabidopsis, rice and *Brassica oleracea* [24], [25], [29]–[31], [33], [34], [39]. The abundance of these transcripts returned to basal levels within 3 d of Pi being re-supplied to the plants (Figure 3AB). The increased expression of two sulphate transporters, SULTR3;1 and SULTR3;4, the latter's transcript abundance increasing 8-fold in Pi deficient leaves 17 d after the withdrawal of Pi from the nutrient solution, was also observed. The increased expression of these sulphur transporters could increase S uptake and/or redistribution within the plant, for incorporation into sulpholipids [31], [67].

The breakdown of phospholipids to release Pi and diacylglycerol groups for sulpho- and galacto-lipids is also essential for the maintenance of functional thylakoid and cellular membranes during Pi deficiency [59]–[62]. The role of phospholipases in the breakdown of phospholipids into their component parts is evident under Pi limiting conditions [31], [65], [66], [68], [69]. Specifically, the role of phospholipase Dζ1 and Dζ2 in the breakdown of phosphatidylcholine under Pi limiting conditions is not only important for lipid turnover, recycling P and providing diacyglcerol for galactolipid biosynthesis [62], [65], [69], but is also important in root development under Pi limiting conditions [64], [69]. In potato leaves, the abundance of transcripts encoding genes with homology to these two phospholipase D isoforms (*PLDζ1* = MICRO.18092.C1; *PLDζ2* = MICRO.15240.C1) increased significantly between 7 and 17 d after the withdrawal of Pi from the nutrient solution, and returned to basal levels after the re-supply of Pi (Figure 3C), consistent with previous observations in other plants [31], [33], [39], [64], [65], [69]. The expression of another two isoforms did not alter significantly during the experiment. Interestingly, the expression of three patatin-like genes (represented by 5 probes) had greater transcript abundance in Pi deficient potato leaves compared to leaves of plants supplied with a full nutrient solution (Figure 3C). Patatins, the main storage proteins in potato tubers [70], also have phospholipase A2 activity [71], [72]. Therefore, there is potential for phosphatidylcholine to be degraded by these enzymes, eventually generating substrates for glycerophosphodiester diesterases. The transcript abundance of one of these glycerophosphodiester diesterases increased by over 120-fold in Pi deficient potato leaves compared to leaves of plants supplied with a full nutrient solution (Figure 3C). This may represent a novel Pi responsive pathway for the recycling of phospholipids in potatoes, and possibly other plant species, the biochemical consequences of which should be determined. Interestingly, the expression of patatin genes is known to be induced by the application of exogenous sucrose [73]. Consequently, the increased abundance of patatin transcripts might be attributed to the accumulation of sucrose under Pi deficiency [45]. Recently, a role for patatins in the regulation of root responses to Pi deficiency, through interactions with auxin, has been proposed [74]. Consequently, there may be a role for these proteins in regulating as well as recycling P in Pi deficient plants.

### Diagnosing Pi deficiency in field grown potato plants

Changes in gene expression in the leaves of Pi deficient potato plants were consistent with previous analyses of transcriptional profiles from Pi deficient leaves of Arabidopsis, rice, *B. oleracea* and *B. rapa* (Table S1; [24], [25], [30], [31], [33], [34], [39]). Since there are commonalities within these transcriptional profiles, both between experiments and between species, we hypothesised that it may be possible to identify a group of genes that are diagnostic for the physiological Pi status of a leaf. To assign the physiological state of a diagnostic potato leaf as coming either from a Pi deficient potato plant or Pi replete potato plant we used class prediction algorithms developed for the classification of cancer tumours [75] to define a set of diagnostic genes and then predict the status of individual leaf samples from field grown potato plants. Class prediction is a supervised learning method where an algorithm uses gene expression profiles from samples of known classification to build a rule to predict the class of samples of unknown classification. The process consists of two steps: First, predictor genes are chosen from a training set of samples with known classification. Second, a cross-validation process tests the ability of the algorithm to correctly classify a set of test samples.

A support vector machine (SVM) algorithm was used to classify the data, since SVM based classification algorithms have been shown to outperform non-SVM algorithms [76]. To classify the data, the SVM attempts to fit a multi-dimensional hyperplane through the data in a way that separates or classifies the individual samples in the data into groups (e.g. Pi replete or Pi deficient). For un-classified samples, the SVM calculates the margin value, which is the distance and direction of the sample from the hyperplane, allowing the SVM to predict which group the sample belongs to [75].

Diagnostic leaves from field grown potato plants were sampled and the RNA extracted, labelled and hybridised to the POCI arrays. The samples were taken from either potato plants supplied with Pi fertiliser or from potato plants that had received no Pi fertiliser. There was a clear effect of Pi fertilisation on shoot [P], with potato plants from unfertilised plots having significantly (*P* = 0.037) lower shoot [P] compared to plants from fertilised plots (Table 2). The shoot [P] from unfertilised potato plants are below the critical P concentration for potato plants and would be considered P deficient [77]–[79]. Biomass accumulation for shoot tissue at tuber initiation and commercial tuber yield at maturity were also significantly (*P* = 0.020 and *P* = 0.033, respectively) reduced in potato plants from unfertilised plots. There was also an effect of Pi fertilisation on shoot [K]; however, the values obtained for both treatments would be considered sufficient for field grown potatoes [77].

**Table 2:** Yield and elemental composition for field grown potatoes plants.

	P fertiliser application			
	unfertilised		fertilised	
	mean	(sem, n = 6)	mean	(sem, n = 3)
Shoot biomass at tuber initiation (g DW plant^−1^)	147.60	(15.90)	218.20	(5.40)*
Shoot [P] (mg P g^−1^ DW)	1.98	(0.10)	5.13	(0.65)*
Shoot [N] (mg N g^−1^ DW)	20.93	(1.07)	24.36	(3.25)ns
Shoot [K] (mg K g^−1^ DW)	60.67	(1.33)	45.87	(4.67)**
Commercial tuber yield (t ha^−1^)	34.93	(3.00)	47.16	(2.20)*

Significance levels are for two sided t-tests, testing for significant differences between the mean values for fertilised and unfertilised treatments.

* = *P*<0.05,

** = *P*<0.01, ns = not significant.

**Figure 4:**
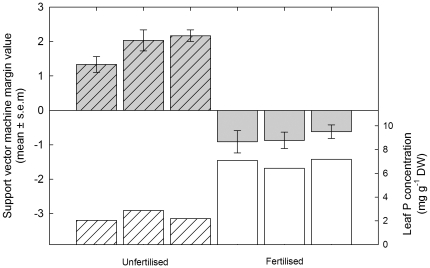
Class prediction for Pi status of Pi fertilised and unfertilised field grown potato plants. Margin values from Support Vector Machine (SVM) analysis of transcriptional profiles of field grown potato plants supplied with (fertilised; plain bars) or without (unfertilised; diagonal lined bars) Pi fertiliser applications (left axis) and the leaf P concentration of potato leaf samples from the same plants (right axis). Margin values are means ± SEM (n = 5).

The gene expression data generated from the diagnostic leaf samples were used as a test set for the groups of diagnostic genes, with the kernel function and sets of diagnostic genes changed systematically to optimise the classification of the field samples (Table 3). Using a set of 200 genes as the diagnostic genes, with any kernel function, successfully characterised all samples correctly. The margin values generated by the SVM indicate the Pi status of the potato plants from individual field plots, and these agree with the shoot [P] data obtained from the same field plots (Figure 4). These data demonstrate the proof-of-concept for the development of diagnostic gene expression markers for shoot Pi status in field grown crops. Additional transcriptional profiles from plants grown at a range of Pi fertiliser application rates and their leaf P concentrations are required to form a robust predictive relationship and further validate this approach.

**Table 3:** Evaluation of groups of diagnostic genes for classifying crop Pi status.

Kernel function	Number of genes used	Actual class	+P	−P	+P	−P	% correct
		Predicted class	−P	+P	+P	−P	
Dot Product (Order 1)	25		5	7	10	8	60.0
Dot Product (Order 2)	25		3	9	12	6	60.0
Dot Product (Order 3)	25		2	7	13	8	70.0
Radial basis	25		2	7	13	8	70.0
Dot Product (Order 1)	50		6	2	9	13	73.3
Dot Product (Order 2)	50		0	3	15	12	90.0
Dot Product (Order 3)	50		0	4	15	11	86.7
Radial basis	50		0	4	15	11	86.7
Dot Product (Order 1)	100		7	0	8	15	76.7
Dot Product (Order 2)	100		0	3	15	12	90.0
Dot Product (Order 3)	100		0	3	15	12	90.0
Radial basis	100		2	3	13	12	83.3
**Dot Product (Order 1)**	**200**		**0**	**0**	**15**	**15**	**100.0**
**Dot Product (Order 2)**	**200**		**0**	**0**	**15**	**15**	**100.0**
**Dot Product (Order 3)**	**200**		**0**	**0**	**15**	**15**	**100.0**
**Radial basis**	**200**		**0**	**0**	**15**	**15**	**100.0**
Dot Product (Order 1)	250		2	0	13	15	93.3
Dot Product (Order 2)	250		1	0	14	15	96.7
Dot Product (Order 3)	250		1	0	14	15	96.7
Radial basis	250		1	0	14	15	96.7
Dot Product (Order 1)	1000		3	0	12	15	90.0
Dot Product (Order 2)	1000		2	0	13	15	93.3
Dot Product (Order 3)	1000		2	0	13	15	93.3
Radial basis	1000		2	0	13	15	93.3
Dot Product (Order 1)	8,663		4	0	11	15	86.7
Dot Product (Order 2)	8,663		2	1	13	14	90.0
Dot Product (Order 3)	8,663		2	2	13	13	86.7
Radial basis	8,663		2	2	13	13	86.7
Dot Product (Order 1)	28,946		2	2	13	13	86.7
Dot Product (Order 2)	28,946		3	1	12	14	86.7
Dot Product (Order 3)	28,946		5	2	10	13	76.7
Radial basis	28,946		5	2	10	13	76.7
Dot Product (Order 1)	42,094		2	9	13	6	63.3
Dot Product (Order 2)	42,094		0	15	15	0	50.0
Dot Product (Order 3)	42,094		0	15	15	0	50.0
Radial basis	42,094		0	14	15	1	53.3

The use of a more refined set of samples from which to identify predictive genes for plant Pi status was also tested to see if the analyses could be improved (data not shown). However, using samples from days 1, 3, 7, and 17 only, it was not possible to identify predictive genes using the Golub method. A set of predictive genes was successfully identified using Fisher's Exact test, but these genes were not successful in classifying the Pi status of field grown crops. It is likely that the reduction in sample numbers from which to identify predictive genes, and the reduced variability in expression profiles within the reduced data set contributed to the poor performance. Consequently, for future development and identification of predictive genes for plant nutritional status, a large data set, covering varying degrees of nutritional status may be necessary.

The 200 genes identified as being diagnostic for potato leaf Pi status contained 136 genes that were also identified as significantly differentially expressed in the leaves of hydroponically grown potatoes after the withdrawal of Pi from the nutrient solution (Table S2). Of the 136 genes, 73% were assigned to clustering groups I, VI, VII and VIII. These groups all contained genes that, on average, had greater transcript abundance in Pi deficient leaves compared to the leaves of plants supplied with Pi throughout the experiment (Table 1). Thus, the majority of genes used to predict the Pi status of potato leaves increased their expression under Pi deficient conditions. Of the 64 genes not previously identified as being differentially expressed in Pi deficient potato leaves, only 13 genes had greater (>1.5-fold) transcript abundance in Pi deficient potato leaves compared to the leaves of plants supplied with Pi throughout the experiment (Table S2). The other 51 genes in this group either had decreased (<0.66-fold) transcript abundance in Pi deficient potato leaves for the majority of time-points, compared to the leaves of plants supplied with Pi throughout the experiment, or their expression levels ranged between 0.66- and 1.5-fold expression in Pi deficient potato leaves compared to the leaves of plants supplied with Pi throughout the experiment. Gene Ontology analysis of these 64 genes identified a greater number of genes that had been annotated with GO terms for phosphoric ester hydrolase activity and glycerol-3-phosphate dehydrogenase (NAD+) activity than would be expected by chance. These included two down-regulated acid phosphatases and a down-regulated NAD dependent glycerol-3-phosphate dehydrogenase (Table S2).

**Figure 5:**
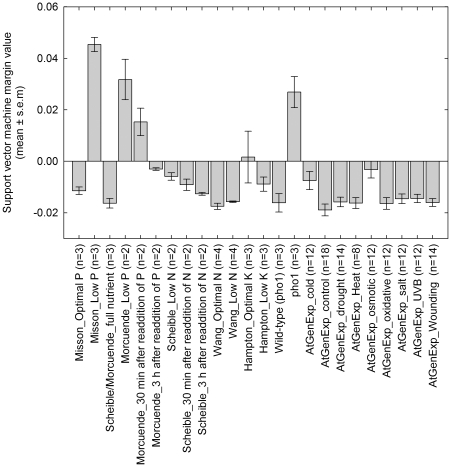
Class prediction of plant Pi status for different nutritional and abiotic stresses. Margin values from Support Vector Machine (SVM) analysis of transcriptional profiles of Arabidopsis plants subjected to nutritional and abiotic stresses [31], [33], [82]–[85]. Arabidopsis orthologs of genes identified as predictive of Pi deficiency in potato were used by a SVM (GeneSpring 7.3) to classify the samples as Pi deficient (positive value) or Pi replete (negative value). Margin values are means ± SEM (n = indicated on x-axis labels). Inset graphs are AtGen Express data for cold and osmotic stress; bars represent mean margin values ± SEM (n = 2).

**Figure 6:**
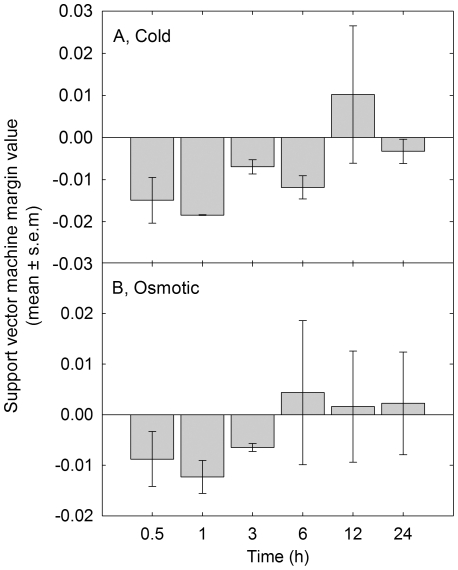
Class prediction of plant Pi status for cold and osmotic stresses. Margin values from Support Vector Machine (SVM) analysis of transcriptional profiles of Arabidopsis plants subjected to (A) cold and (B) osmotic stresses over 24 h [85]. Arabidopsis orthologs of genes identified as predictive of Pi deficiency in potato were used by a SVM (GeneSpring 7.3) to classify the samples as Pi deficient (positive value) or Pi replete (negative value). Margin values are means ± SEM (n = 2).

To further validate the robustness of the genes identified in potato for predicting crop Pi status, their ability to predict the Pi status of plants under Pi deficiency and other abiotic stresses was tested. Data from Arabidopsis were used, since there are large collections of microarray data available for Arabidopsis plants subjected to various nutrient and abiotic stresses [80]. Arabidopsis orthologs for the 200 predictive potato genes were identified using translated Blast analyses between the potato gene sequences used to design the probes on the array and Arabidopsis protein sequences [81]. A total of 148 Arabidopsis genes were identified with significant homology to the 200 predictive potato genes (Table S2). The 148 genes were used to predict the Pi status of Arabidopsis plants subjected to various abiotic stresses, or mutants with known shoot Pi phenotypes (Figure 5). The margin values generated by the SVM indicate whether the sample is likely to be Pi deficient (positive margin value) or Pi replete (negative margin value). The Pi statuses of Arabidopsis leaves grown with or without Pi were correctly predicted based on the transcriptional profiles of these samples (Figure 5; [31], [33]). Following the re-addition of Pi to Pi deficient plants, the margin values decrease from 0.032 to 0.015, 30 min after re-addition, then to −0.003, 3 hours after re-addition of Pi, suggesting they are now Pi replete and demonstrating the rapid response of plants to the re-supply of Pi (Figure 5). All samples subjected to N deficiency and re-addition were all classified as Pi replete [82], [83]. The transcriptional profile for the Arabidopsis mutant *pho1*, which has a significantly reduced shoot [P] [84], was correctly classified as Pi deficient. For non-nutritional abiotic stresses, the SVM classified them all as Pi replete across the abiotic stress AtGen Express transcriptional profiles [85]. Interestingly, as cold and osmotic stress developed in these plants the plants became more Pi deficient as classified by the SVM (Figure 6). Cold and osmotic stress could affect the availability and uptake of Pi from the growth media and alter the Pi status of the plant. Indeed, there are overlaps with the transcriptional profiles of plants under cold and osmotic stress and Pi deficient plants [24], [86].

### Conclusions

Our data demonstrate the potential for using a focused group of genes, identified as being diagnostic for Pi deficiency, for determining the Pi status of a field grown potato crop. This proof-of-concept demonstrates the potential for using such techniques to identify biotic and abiotic stresses in crop plants under field conditions. The use of many diagnostic genes should ensure the assay is robust against other environmental stimuli, which could alter the expression of one or more of these genes. This was demonstrated using data from Arabidopsis subjected to a range of nutritional and abiotic stresses. More trials are needed to validate the predictive abilities of these genes across a wider set of field conditions and soil types. Further optimisation is also required against a range of Pi fertiliser application rates to determine the sensitivity of predictions made using this method and also the impact of different potato varieties on hybridisation efficiencies with the probes of the diagnostic genes. Since many biotic and abiotic stresses can also be characterised in terms of their transcriptional profiles, this technique has the potential to screen crop plants for many stresses simultaneously.

Given the speed with which these assays could be conducted (2–3 d) and the reduction in costs of performing microarray experiments over time, these assays may have the potential in the future to provide rapid diagnosis and allow remedial Pi fertiliser applications, most likely as foliar applications, to minimise yield losses from Pi deficiency. Ultimately, optimisation of Pi fertiliser applications will reduce their potential negative impacts on local environments and water quality, and the depletion of non-renewable natural resources.

## Materials and Methods

### Plant material and glasshouse growth conditions for monitoring the response of potato plants to Pi availability

*Solanum tuberosum* var. Kennebec micro-plants (Higgins Agriculture, Doncaster, UK) were initially transferred to rockwool blocks (3.5×3.5×4 cm; Grodan, Hedehusene, Denmark) and watered with de-ionised water in plastic trays. The plastic trays with plants were covered with plastic film to maintain humidity, and placed in a growth room at 25°C. Once established (7 d), rockwool blocks were transferred to a nutrient film technique (NFT) hydroponic system in a glasshouse [87]. The experiments were carried out between May 2003 and June 2004 in a 40 m^2^ glasshouse compartment at the University of Warwick (Wellesbourne, UK; latitude 52°12′32″N, longitude 1°36′06″W, 46 m above sea level). Daylight was supplemented by artificial lighting (Son-T 400-W Philips phi 0.85i) to maintain 16 h light d^−1^. The glasshouse was set to maintain temperatures of 20°C by day and 15°C at night using automatic vents and supplementary heating. The NFT system comprised 12 individual gullies. Each gulley was covered by a PVC cover with 72 holes. The gullies were spaced 0.26 m apart (centre-to-centre) in two groups of six within the same glasshouse compartment. Each gully was connected to one of two water-storage tanks that each contained 200 L of deionised water to which mineral nutrient salts were added. The nutrient solutions contained 2 mM Ca(NO_3_)_2_, 2 mM NH_4_NO_3_, 0.75 mM MgSO_4_, 0.5 mM KOH, 0.25 mM KH_2_PO_4_, 0.1 mM FeNaEDTA, 30 µM H_3_BO_3_, 25 µM CaCl_2_, 10 µM MnSO_4_, 3 µM CuSO_4_, 1 µM ZnSO_4_, 0.5 µM Na_2_MoO_4_. Nutrient solutions were adjusted daily to pH 6, using H_2_SO_4_, and solutions were replaced completely twice a week. All nutrients were supplied in excess to prevent deficiency symptoms occurring for other nutrients. Samples of nutrient solutions were analysed for elemental composition before and after nutrient solutions were changed. To induce Pi deficiency, the Pi supplied as KH_2_PO_4_, was replaced with K_2_SO_4_ to supply the same concentration of K.

Once the plants were established in the NFT system, the nutrient solution supplying half of the plants was replaced with one containing no P, whilst the nutrient solution supplying the remaining plants was maintained as a full nutrient solution. The no Pi treatment was imposed at 10:00 am on day 0, and samples for microarray analyses were taken at 12 noon, 2 h after the start of the treatment. All samples were taken at 12 noon, midway through the photoperiod to avoid detection of circadian induced changes in gene expression between time points. When changes in nutrient solution coincided with sampling points for microarray analysis for the remainder of the experiment, nutrient solutions were changed after samples were taken to avoid changes in gene expression associated with changing the nutrient solution. Shoots of plants were harvested destructively during the course of the experiment. At each harvest, diagnostic leaves, defined as the youngest fully expanded leaves, taken as the second leaf below the crown of the plant [50], from three plants supplied with a full nutrient solution and three plants supplied with a nutrient solution containing no Pi were sampled and snap frozen in liquid nitrogen before RNA extraction. The remaining shoot tissue was harvested and fresh weights (FW) of leaf and stem tissue recorded. Sample dry weights (DW) were recorded after conventional oven-drying at 80°C for 72 h. Dried samples were subsequently milled. Total leaf P and K concentrations were determined using the micro Kjeldahl method, in which a 0.1 g subsample of dried leaf material was digested for 1 h, after the addition of 1 mL of H_2_O_2_ and 2 mL of a H_2_SO_4_/Se catalyst [88]. Inductively-coupled plasma emission spectrometry (JY Ultima 2, Jobin Yvon Ltd., Stanmore, Middlesex, UK) was used to determine elemental tissue concentrations. To determine tissue N concentrations, an aliquot of dried tissue sample was loaded directly into a combustion analyzer (CN 2000, LECO UK, Stockport, Cheshire, UK) and analyzed for percentage N via an internal thermal conductivity detector. As plants were sampled during the experiment, the resulting gaps in the PVC strips were immediately covered to maintain humidity and to reduce algal growth in the gullies. Potato plants supplied with a nutrient solution containing no Pi were grown for 28 d, after which Pi was re-supplied, and the plants grown for a further seven days. Control plants received a full nutrient solution throughout the experiment.

Shoot biomass and tissue elemental composition data were analysed using GenStat (9^th^ Ed., VSN International Ltd, UK) to determine predicted means and standard errors for individual time points and treatments. Data were log transformed and summarised using REML analysis, with a fixed model as [days after Pi withdrawal*treatment] and a random model of [Block/Row/Plant]. Predicted means were back transformed and standard errors were back transformed and converted to 95% confidence intervals.

### Plant material and field growth conditions for potato plants

Seed potatoes (*Solanum tuberosum* var. Kennebec; Higgins Agriculture Ltd) were placed in chitting trays for two weeks before planting. Seed tubers had a mean dry weight of 29 g plant^−1^ (±2 SEM, n = 20). Seed tubers were planted into established phosphate-fertiliser treated plots on a field site of low P-status at Wharf Ground, Warwick-HRI [89]. Fertilised plots were established using triple super-phosphate (TSP). The availability of soil Pi in each plot was adjusted by the addition of TSP to give an average Olsen's P/bicarbonate extractable Pi of 150 mg L^−1^ [90]. Unfertilised plots had an average Olsen's P/bicarbonate extractable Pi of 25 mg L^−1^. Irrigation was supplied when required using oscillating lines, and pesticide and herbicide applications were made according to agricultural best practice. The field received annual fertiliser applications of 185 kg ha^−1^ N, supplied as NH_4_NO_3_, 325 kg ha^−1^ K_2_O, supplied as K_2_SO_4_ (also supplying 292 kg ha^−1^ SO_3_) based on soil analysis results and UK fertiliser recommendations for a long haulm longevity potato crop [91].

Seed potatoes were planted on the 12 April 2006. All samples were taken at a constant time within the photoperiod, at noon. Diagnostic leaves, defined as the youngest fully expanded leaves (taken as the second leaf below the crown of the plant; [50]), were taken from 15 plants on plots that had received Pi fertiliser and from 15 plants on plots that had received no Pi fertiliser on 30 June 2006, 79 d after sowing. All tissue samples were snap frozen in liquid nitrogen in the field at harvest and subsequently ground under liquid nitrogen and stored at −80°C before RNA extraction.

### RNA extraction

RNA was extracted from tissue samples using methods described previously [92]. To each sample, 1 mL of TRIzol reagent was added, and total RNA was subsequently extracted according to the manufacturer's instructions (Invitrogen, Paisley, UK), with the following modifications: (i) after homogenisation with the TRIzol reagent, the samples were centrifuged to remove any remaining plant material, and the supernatant was then transferred to a clean Eppendorf tube and, (ii) to aid precipitation of RNA from the aqueous phase, 0.25 mL of isopropanol and 0.25 mL of 1.2 M NaCl solution containing 0.8 M sodium citrate were added. This procedure precipitated the RNA whilst maintaining the proteoglycans and polysaccharides in a soluble form. Extracted total RNA was then purified using the ‘RNA Cleanup’ protocol for RNeasy columns with on-column DNase digestion to remove residual chromosomal DNA (Qiagen, Crawley, West Sussex, UK). Samples of total RNA were checked for integrity and quality using an Agilent Bioanalyser (Agilent Technologies, Santa Clara, CA, USA).

### Experimental design, hybridisation and scanning of microarrays

To monitor the maximum number of transcripts from potato and determine transcriptional changes under Pi deficiency the oligonucleotide potato microarray developed by the international Potato Oligo Chip Initiative (POCI) was used. The POCI array is an Agilent oligonucleotide array representing 42,034 potato sequences [40]. This platform was used to identify potato genes that respond specifically to Pi deficiency and to monitor the expression of diagnostic markers to predict the Pi status of potato plants grown under field conditions and supplied with different amounts of Pi fertiliser.

A full dye swap model was used to determine the expression profiles of potato leaves after the withdrawal of Pi from the nutrient solution. All samples were independently labelled with Cyanine 3 and Cyanine 5 and combined in reciprocal combinations on the array. Three biological replicates for control and treated plants at each time point were analysed, generating a total of 42 samples, which were hybridised to 84 arrays to include full dye swaps. For field grown test samples, a one-colour model was used with samples labelled with Cyanine 3. Fifteen samples from Pi fertilised and 15 samples from unfertilised plots were hybridised to separate arrays. Labelled cRNA samples were generated from RNA samples using the Low RNA Input Fluorescent Linear Amplification Kit according to the manufacturer's instructions (Agilent Technologies). First, complementary DNA (cDNA) was generated from total RNA, using 3 µg of total RNA and 5 µL of T7 promoter primer. Then cRNA was synthesised from the double-stranded cDNA using T7 RNA polymerase, incorporating Cyanine 3- or Cyanine 5-labelled CTP fluorescent dyes (PerkinElemer Life and Analytical Sciences, Boston, MA, USA). Labelled cRNA samples were cleaned using the ‘RNA Cleanup’ protocol for RNeasy columns (Qiagen) performed at 4°C and eluted using two 30 µL volumes of nuclease-free H_2_O. Mean dye incorporation for labelled cRNA was 15.65 (±0.28 SEM, n = 42) pmol dye µg^−1^ cRNA.

Hybridisation cocktails were prepared using the *In situ* Hybridisation Kit according to the manufacturer's instructions (Agilent Technologies). To each cocktail, between 0.5 and 3 µg of labelled cRNA per sample was used. Hybridisation cocktails were hybridised to the microarrays in a hybridisation oven (Agilent Technologies) at 65°C for 17 hours. Microarrays were rotated at 10 rpm during the hybridisation procedure to ensure even hybridisation of the sample across the microarray. After hybridisation, microarrays were transferred to a slide rack in a staining dish. The slides were washed in staining dishes with magnetic stirring bars to aid washing in (i) 6× SSPE solution containing 0.005% N-lauroylsarcosine for 1 min, (ii) 0.06× SSPE solution containing 0.005% N-lauroylsarcosine for 1 min, (iii) acetonitrile for 1 min, and (iv) a stabilisation and drying solution (Agilent Technologies) for 30 s.

Microarrays were scanned on a DNA Microarray Scanner BA (Agilent Technologies). The Extended Dynamic Range function was used to scan the microarrays, and data extracted from the scanned images using the Feature Extraction software package. Feature extraction was performed using a spatial de-trend algorithm within the software package. The output files from the Feature Extraction software provide several estimates of signal intensities for each probe. For further analysis the ‘processed’ signal values were used. These values had been adjusted for spatial variation across individual arrays, and normalised using a global background subtraction and a Lowess normalisation. Microarray scans were checked for quality using data from the Feature Extraction software and distribution of data using GeneSpring GX analysis software (v 7.3; Agilent Technologies). Data not meeting these criteria were discarded and the hybridisations repeated. All data was curated to MIAME compliant standards and the raw data files were deposited in the MIAME compliant database, Array Express, under accession numbers E-MTAB-629 and E-MTAB-634.

### Microarray data analysis

The processed signal values were imported into GeneSpring GX. Data from individual microarrays were subjected to a Lowess normalisation in which a Lowess curve was fitted to the log-intensity versus log-ratio plot. The Lowess fit at each point was calculated using 35% of the data. This curve was used to adjust the control value for each measurement. If the control channel was lower than 10 then 10 was used instead. Each gene was divided by the median of its measurements in all samples for an experiment. Data from the Pi response time course were pre-filtered by 1) removing genes whose raw signal value was less than 50 in five of the seven time points, 2) removing genes flagged as absent and 3) removing genes whose normalised signal value remained between 0.8 and 1.2 at all time points to leave 28,946 genes for further analysis.

To identify significantly differentially expressed genes between treatments an ANOVA with a Benjamini & Hochberg FDR multiple testing correction was used. Gene Ontology terms assigned to genes were analysed using the Gene Ontology Browser in GeneSpring GX.

### Class prediction analysis

For class prediction, the support vector machine implemented in the Class Prediction tool of GeneSpring GX was used to classify the data. Sets of diagnostic genes were selected using the Golub method with the Polynomial Dot Product (Order 1) kernel function. Different kernel functions and sets of diagnostic genes were changed systematically to optimise the classification of samples. The training set used to predict diagnostic genes was a modified version of the dataset obtained from the Pi withdrawal and re-supply experiment, in which the two-colour data were treated as one-colour data to allow samples to be classed as being Pi replete or Pi deficient, generating 84 samples. All samples from day 0 and day 31, and those supplied with Pi for the duration of the experiment were classed as Pi replete (+P). Samples from day 1 to day 29 that were supplied with no Pi were defined as Pi deficient (−P). Sets of diagnostic genes were selected using the Golub method with the Polynomial Dot Product (Order 1) kernel function. The 84 glasshouse samples monitoring the transcriptional Pi response of potato plants, were used as the training set and the test set to generate sets of diagnostic genes containing 25, 50, 100, 200, 250, 1000, 8,663, 28,946 and 42,094 genes. The ability of these sets of genes to classify the expression profiles from leaves of field grown potato plants, which had been supplied with or without Pi fertiliser, as being Pi deficient or Pi replete was then tested.

Raw data (cel files) from Arabidopsis nutritional and abiotic stress Affymetrix Arabidopsis ATH1 GeneChip arrays were all downloaded from NASC arrays (http://affymetrix.arabidopsis.info/narrays/experimentbrowse.pl), with the exception of data from [31] which was downloaded from Array Express (http://www.ebi.ac.uk/microarray-as/ae/; E-MEXP-791) and data from [33], [82], which were provided by WR Scheible (personal communication; Max Planck Institute for Molecular Plant Physiology). All data were loaded into GeneSpring and pre-processed using RMA normalisation. Arabidopsis orthologs of the 200 potato predictive genes were identified using tblastx [81], which identified 148 Arabidopsis orthologs. These genes were used in the SVM in GeneSpring class prediction tool to classify the samples as Pi deficient of Pi replete.

## Supporting Information

Figure S1 Hierarchical clustering of 1,659 genes significantly (*P*<0.01) differentially expressed by more than 1.5 fold at two or more, of the seven time points (Table S1). Hierarchical clustering was performed using Cluster [42] and visualised using Maple Tree (http://rana.lbl.gov/EisenSoftware.htm).
(TIF)

Table S1 Normalised signal values and annotation data for 1,659 genes significantly (*P*<0.01) differentially expressed by more than 1.5 fold at two or more, of the seven time points.
(XLSX)

Table S2 Normalised signal values and predictive power of 200 genes identified by a support vector machine algorithm for the class prediction of the physiological Pi status of field grown potato leaves.
(XLSX)
