# Supplementary figures and images for: Gene Expression Changes in Phosphorus Deficient Potato (Solanum tuberosum L.) Leaves and the Potential for Diagnostic Gene Expression Markers
Source: PLoS One. 2011 Sep 14;6(9):e24606. doi: 10.1371/journal.pone.0024606 (PMC3173461; doi:10.1371/journal.pone.0024606)

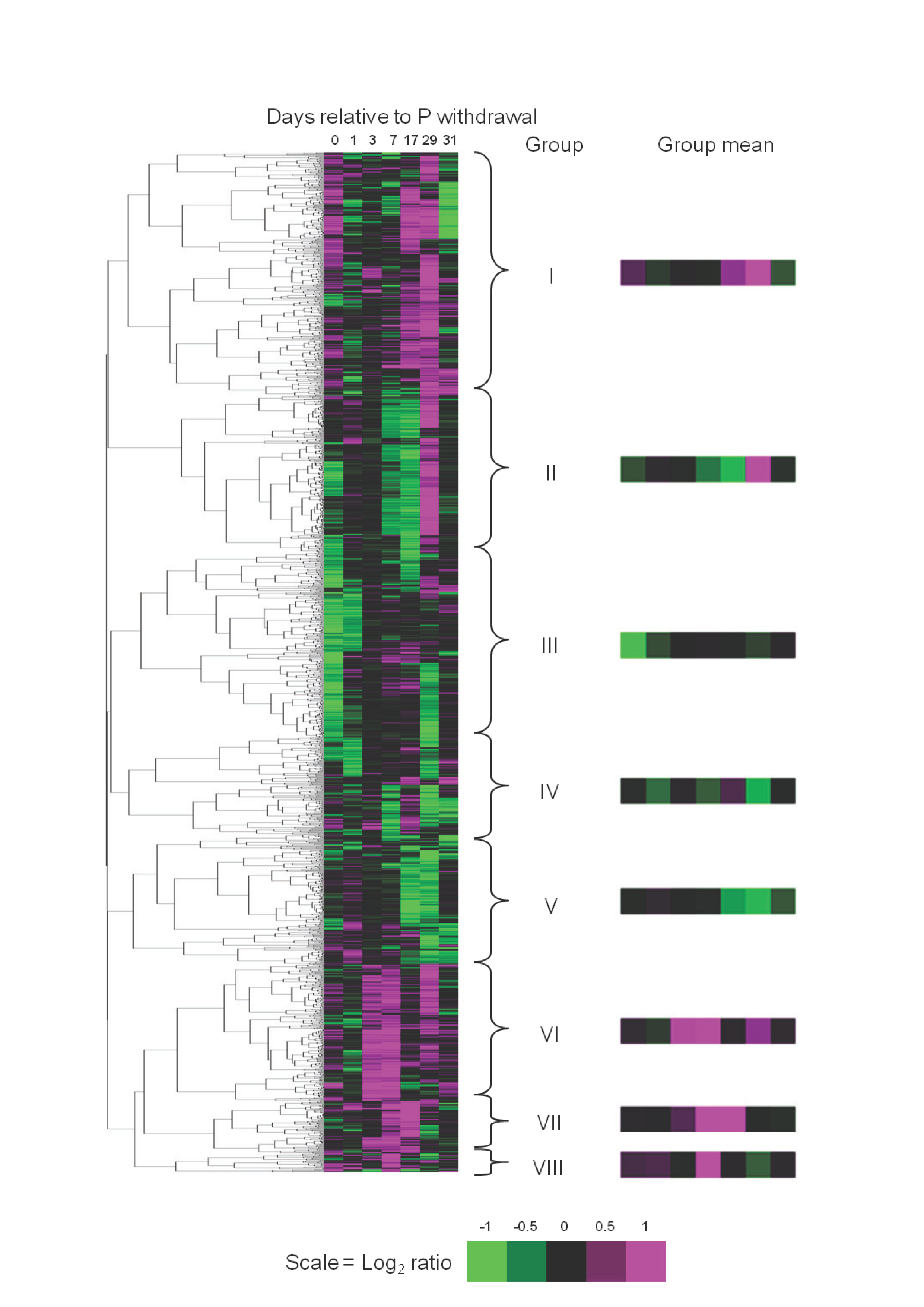

Supplement: Figure S1 — Hierarchical clustering of 1,659 genes significantly (P<0.01) differentially expressed by more than 1.5 fold at two or more, of the seven time points (Table S1). Hierarchical clustering was performed using Cluster [42] and visualised using Maple Tree (http://rana.lbl.gov/EisenSoftware.htm). (TIF) [file pone.0024606.s001.tif]
